# Giant diffuse cerebellar AVM: managing ultimate intraoperative challenges

**DOI:** 10.3171/2020.10.FOCVID2093

**Published:** 2021-01-01

**Authors:** Benjamin K. Hendricks, Aaron A. Cohen-Gadol

**Affiliations:** 1 *The Neurosurgical Atlas*, Carmel; and; 2 Department of Neurological Surgery, Indiana University, Indianapolis, Indiana

**Keywords:** arteriovenous malformation, neurovascular surgery, posterior cranial fossa

## Abstract

Surgery within the posterior cranial fossa uniquely requires excellence in microsurgical technique, given the complexity of the neurovascular structures housed within this region. Arteriovenous malformations (AVMs) within this region represent the greatest surgical challenge because of the difficulty in resecting an AVM completely while preserving the highly eloquent surrounding structures. The AVM in this video exemplifies a surgeon’s “most challenging case,” a surgery that spanned two stages, including 14 hours of resection, but concluded with complete resection despite the complexity of deep arterial and dural feeders.

The video can be found here: https://youtu.be/WNBuwFHSrQ0

## Transcript

**0:20 Introduction.** Hello, ladies and gentlemen. This video describes technical nuances for getting out of trouble while operating on giant AVMs, and specifically those in the posterior fossa.

**0:33 Case Description.** This is a 43-year-old female who presented with progressive unsteadiness, and MRI evaluation demonstrated a very large, more than 6-cm left-hemispheric malformation with both superficial and deep draining veins involving part of the medial structures as well. You can see the extent of the malformation. Angiogram again demonstrates very diffuse malformation involving the hemisphere with feeding vessels from the PICA, AICA, and superior cerebellar arteries. Here you can see the later phases of the angiogram as well as the deep draining veins.

**1:17 Postembolization Angiography.** Here you can see after embolization where some of the branches of the superior cerebellar artery that are more difficult to reach were embolized more selectively. Again, a fair amount of niditis still is present. An external injection also demonstrated fair amount of contribution from the external carotid arteries.

**1:44 Patient Positioning and Incision.** Here you can see the positioning for this patient. A hockey-stick incision was used in the lateral position. A left-sided suboccipital craniotomy was conducted.

**1:55 Dural Opening and Nidus Exposure.** Upon opening the dura, as we expected, we encountered a fair amount of bleeding from the meningeal branches. These were controlled efficiently using wide-tip bipolars. Next, the dura was opened generously. You can see the malformation. We started from exposing the deep portion of the cerebellopontine angle cisterns to protect the lower cranial nerves. The PICA, which was very hypertrophied, was identified early on, and the feeding vessels to the malformation were coagulated early on to devascularize the malformation further. Here is the step for identification of the brainstem. Another feeder going to the malformation that is being embolized at this step, or coagulated. Next, we continue to devascularize the malformation more inferiorly. Here is along the CP angle where the feeding vessels from AICA or PICA are further devascularized while protecting the lower cranial nerves.

**3:05 Encounter of Delicate Feeding Vessels.** As you can see, some of these feeding vessels can be quite fragile, and persistence is required to control the bleeding. We continue devascularization further inferiorly, and then here you can see the vertebral artery that was identified early on. Again, the principle is to identify the critical structures early, so if you run into bleeding you can control them and protect them. Here you can see the disconnection more superiorly. Again, as we went deeper into the cerebellum, the deep white matter bleeding was quite challenging. It’s critical to be patient, to remove more brain until you’re farther away from the nidus to achieve hemostasis, especially in the cerebellar AVMs.

**4:02 Circumferential Dissection With Significant Bleeding.** Here you can see continuation of the bleeding as the malformation is being devascularized. We wanted to stay away from the deep nuclei, and as you can see, we’ve come close to the nidus and therefore further bleeding was encountered. We used a combination of removing a little bit of cerebellum to expose more of the normal walls of the deep white matter feeders and sometimes gentle tamponade to be able to control bleeding. Here again, continuation along the CP angle to be able to devascularize the AVM from the ICA branches. Some of these turned out to be quite difficult to control. However, with persistence, we were able to control those, again protect the branches of the vertebral artery, after which we divert our attention to more of the superior aspect of the CP angle and again moved around the malformation as much as possible. Here you can see torrential bleeding just around the superior petrosal vein, and at some point the bleeding was unsurmountable and excessive, and we had to even use tamponade with my finger to be able to control the bleeding and a piece of cotton soaked in thrombin, after which a retractor was used to hold the thrombin-soaked cotton in place. This maneuver is effective to be able to control the bleeding from the venous sinuses that are within the tentorium. After controlling this, again we knew that the dominant vein, which is more medial, is protected at all times. Here again, going circumferentially around the malformation and devascularize the lesion. We knew that this AVM is going to be removed in two stages and not one. Therefore, as many of the feeding vessels were taken and draining veins protected. Here again, you can see working along the CP angle, trying to devascularize the malformation, just along the CP angle. Unfortunately, we ran into a very engorged vein that exploded. In this case, I recommend using thrombin-soaked cotton to gently tamponade the vein for it to thrombose. Aggressive coagulation can lead to more explosion of the vein and disruption of its wall and further bleeding.

**6:56 Closure of Stage 1.** We continued again and felt that at this junction, we achieved adequate hemostasis to complete stage 1 of the operation. Therefore, following immaculate hemostasis, the nidus was left in place. Further inspection of the structures at depth of the nidus was performed.

Here again, it’s the end of step 1. The bone flap was not replaced. Unfortunately, the postoperative CT scan demonstrated some hematoma within the CP angle. However, the exam of the patient was relatively good in terms of being just slightly hemiparetic on the left side but awake, and as you can see here, is the bleeding in the area of the CP angle, and here is the angiogram just before stage 2 that demonstrated residual malformation along the inferior aspect of the cerebellum.

**8:02 Beginning of Stage 2.** This operation was staged in order to allow the rest of the normal cerebellum to adjust more slowly and gradually to the resection of the arteriovenous malformation and regional changes in perfusion. In addition, we would like to decrease the significant blood loss in one operation. Of course, there are risks with staging this surgery and resection of the AVM, as this can lead to potentially undesirable hemorrhage from the nidus between the two operations. The blood pressure was very much controlled between the two operations, and the systolic blood pressure was strictly kept at < 120 mm Hg during this time.

Here’s the reopening during stage 2 about a week later, where you can see the thrombosed vein along the CP angle, which was compressive, and after the nidus was removed, we’re able to get into the vein and remove some of the clot within the vein to decompress the brainstem. Next, we divert our attention to the superior aspect of the cerebellum, where the residual malformation was noted on the preoperative angiogram. Here is one of the draining veins that was coagulated after the residual malformation was removed. Here again, moving around the CP angle, making sure that there’s no other compressive lesion. Again, we remove the clot within the vein to decompress the brainstem and after which intraoperative angiogram demonstrated complete removal of the malformation, and this patient had some left-sided hemiparesis and facial weakness that improved significantly at 6 months.^1–5^ Thank you.

## Author Contributions

Primary surgeon: Cohen-Gadol. Editing and drafting the video and abstract: both authors. Critically revising the work: both authors. Reviewed submitted version of the work: both authors. Approved the final version of the work on behalf of both authors: Cohen-Gadol. Supervision: Cohen-Gadol.
